# Rapid Liquid Recognition and Quality Inspection with Graphene Test Papers

**DOI:** 10.1002/gch2.201700037

**Published:** 2017-07-10

**Authors:** Xin Jiang, Tingting Yang, Changli Li, Rujing Zhang, Li Zhang, Xuanliang Zhao, Hongwei Zhu

**Affiliations:** ^1^ State Key Laboratory of New Ceramics and Fine Processing School of Materials Science and Engineering Tsinghua University Beijing 100084 China; ^2^ Center for Nano and Micro Mechanics Tsinghua University Beijing 100084 China

**Keywords:** electronic tongue, graphene, liquid sensing, nanocomposite

## Abstract

Electronic tongue is widely applied in liquid sensing for applications in various fields, such as environmental monitoring, healthcare, and food quality test. A rapid and simple liquid‐sensing method can greatly facilitate the routine quality tests of liquids. Nanomaterials can help miniaturize sensing devices. In this work, a broad‐spectrum liquid‐sensing system is developed for rapid liquid recognition based on disposable graphene–polymer nanocomposite test paper prepared through ion‐assisted filtration. Using this liquid‐sensing system, a number of complex liquids are successfully recognized, including metal salt solutions and polymer solutions. The electronic tongue system is especially suitable for checking the quality of the foodstuff, including soft drinks, alcoholic liquor, and milk. The toxicants in these liquids can be readily detected. Furthermore, the novel material‐structure design and liquid‐detection method can be expanded to other chemical sensors, which can greatly enrich the chemical information collected from the electrical response of single chemiresistor platform.

## 1 Introduction

Flexible electronics are the cornerstone of wearable devices, and have many potential applications in mobile healthcare, smart city, and other fields.^1^, ^2^ The need for rapid qualitative analysis of chemical environments has fostered the research on artificial bionic senses. Flexible and portable devices that can detect and identify liquids, such as foodstuff, organic solvents, biological samples, etc., allow noninvasive monitoring and are thus important for mobile healthcare.^3^ The Internet of things also requires the rapid detection of all kinds of chemical for quality monitoring.^4^, ^5^ Therefore, a flexible liquid‐sensing device is essential for meeting the future needs in various aspects of liquid monitoring.

Conventional technologies of liquid sensing includes chemical analysis, spectroscopic analysis, liquid parameter analysis, bioelectronic/electronic tongue and chemiresistor. Chemical analysis method has a long history, e.g., titrimetry analysis and colorimetric analysis. As for it is dependent on a specific chemical reaction, it is targeted detection and could achieve qualitative/quantitative analysis.^6^ Spectroscopic analysis instruments, such as X‐ray, photonic‐crystal fibers, surface‐enhanced Raman spectroscopy, could realize accurate chemical analysis.^7^, ^8^, ^9^, ^10^, ^11^ However, chemical and spectrum analysis method requires expensive laboratory equipment and highly qualified personnel, which are not suitable for real‐time on‐the‐spot detection. Liquid parameter analyzers, such as surface tension apparatus or viscometer, are accurate, fast, and portable,^12^ but its application was limited to the identification of organic solvents or specific liquids.

Electronic tongue consisting of an array of nonselective chemical sensors could analyze simple or complex solutions quantitatively or qualitatively.^13^, ^14^, ^15^ Electronic tongues based on different principles have been proposed, including voltammetric, potentiometric, impedimetric, optical, and enzymatic sensors.^14^, ^16^, ^17^, ^18^, ^19^, ^20^ Owing to structural limitations, existing electronic tongues could hardly be embedded into flexible, portable, and wearable devices. Bioelectronic tongues obtain complex chemical information in biomaterial platform, including nanovesicles or living cells.^21^, ^22^, ^23^ With high‐performance odorant discriminatory ability in mixtures, bioelectronic tongues are suitable for portable rapid detection equipment, but the preparation cost is relatively high, and the testing conditions are complicated. Chemiresistor could change electrical resistance in the presence of chemical, which has widely applications in gas sensor.^24^ Chemiresistor is characterized by high detection accuracy, relatively simple detection conditions, low manufacturing cost. However, in chemiresistor‐based liquid‐sensing system, the mechanism is based on the swelling of polymer substrate, so the detected liquid only limited to organic solvents.^25^, ^26^, ^27^ As for its relatively simple structure, chemiresistor could be easily integrated as part of a flexible functional circuit. With designed sensing materials, electrical tongue based on chemiresistor has great potential to offer a unique and compelling capability for classification and detection of tested liquid.

Carbon materials, such as carbon nanotube network^28^ and graphite,^29^ can respond to chemicals and have shown great potential in various areas. Graphene, a well‐known 2D carbon nanomaterial, has high current density, outstanding mechanical and optical properties, as well as many other extraordinary properties.^30^ Therefore, graphene has a wide range of applications in functional flexible devices, including flexible energy conversion/storage devices,^31^, ^32^ chemical/strain sensors.^33^, ^34^ Some researchers reported that graphene‐based chemiresistors could analyze volatile organic compounds (VOCs)^4^, ^35^, ^36^ and humidity.^37^, ^38^ However, the application of graphene in liquid sensing was very limited, and the graphene‐based liquid‐sensing devices could only recognize a small number of solvents, such as water, ethanol, isopropanol (IPA), and acetone. For identification of complex solutions, graphene was only applied as conductive material, and the sensor was based on swelling polymer^27^, ^39^ or biological materials,^21^, ^40^ such as DNA or living cells. The main difficulty in constructing a graphene‐based broad‐spectrum liquid‐sensing chemiresistor was information complexity. In previous studies, the electrical response obtained by graphene sheets in presence of complex solutions was single‐channel response, and it was difficult to analyze enough information to distinguish similar liquids.

In this work, we introduce a flexible and portable graphene‐cellulose nanocomposite test paper (NCTP) comprised of graphene lamellar membrane on a polymer substrate that could qualitatively analyze simple and complex solutions with partial specificity. In the sensing process, the liquid droplet would penetrate into NCTP, impact the contact state of the graphene flakes and the carrier density of the graphene sheet, and alter the total resistance. The relative resistance was depended on the properties of the liquid, such as polarity, viscosity, and vaporization heat. We proposed a recognition algorithm applied to analyze the electrical response of graphene‐based chemiresistors. Descriptive parameters were extracted from the resistance curve to give the performance data, the dimension of the data structure was reduced by principal component analysis (PCA), and the pattern recognition classifier of the electrical signal is based on support vector machine. The NCTP‐based liquid‐sensing system was suitable for the qualitative and quantitative analysis of various liquids, including water and VOCs. We successfully tested a number of organic solvents, as well as the aqueous solutions of polymers and metal salts. The NCTP was also designed to assess the quality of milk and liquor, and detect their counterfeits. Further utilization of NCTP in complicated tasks for industrial and environmental analysis is also possible. Because of its good selectivity, rapid response and flexibility, the disposable NCTP may have promising application in making versatile, flexible, and portable liquid‐sensing devices for inactive healthcare, mobile beverage quality test, and environmental monitoring.

## 2 Results and Discussion

### 2.1 Structural Design and Liquid‐Sensing Mechanism of NCTPs

**Figure 1:**
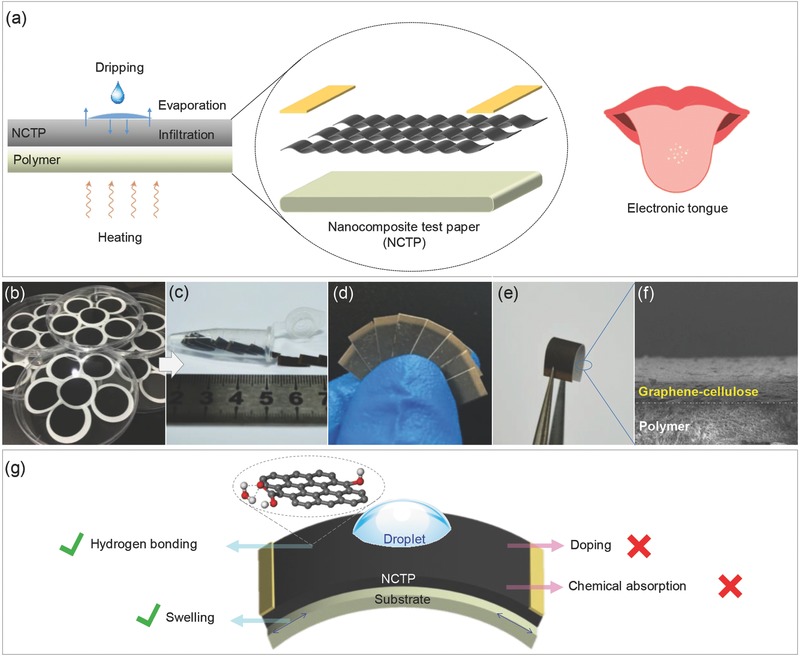
a) Schematic diagrams for test procedure and NCTP structure working as an electrical tongue. b) As‐prepared nanocomposite films and c–e) NCTPs. f) Side‐view SEM image of NCTP (scale bar: 10 μm). g) Liquid‐sensing mechanism.

Liquid sensing by the NCTP system works as illustrated in **Figure** **1**a (left panel). The liquid is dropped on the NCTP surface, infiltrates into the conductive graphene‐assembled film through the micropores, and then evaporates. The electrical conducting properties of NCTP vary when the physical state of the droplet changes. The graphene film has been designed to bear microchannels to allow the liquid to pass, and has a suitable thickness (≈5 μm) that ensures sufficient contact between the liquid and the graphene sheets. Hence, the liquid droplet can be adequately retained on the film and slowly evaporate as desired.

As shown in Figure 1a, the structure of the NCTP is a much less complicated than that of most other types of liquid and gas‐sensing devices.^16^, ^18^, ^23^, ^40^ The film was prepared from sodium carboxymethylcellulose (CMC)‐modified graphene conductive ink through vacuum filtration (Figure 1b). The synthesis was rapid and simple, and was easily implemented in batch. The resulting wafer‐scale graphene film was flexible, and had a relatively smooth and dense surface (Figure 1c–f). More detailed information about the structure of the NCTP is given in Figure S1 (Supporting Information). The NCTP was prepared from the graphene film by first cutting the film and then depositing the electrodes. Because the NCTP was small and flexible, it could easily build lightweight and portable liquid‐sensing devices. In the NCTP, the graphene sheets were densely packed on the polymer filter membrane and overlapped with each other. The surface of the NCTP had some voids to facilitate the penetration of the liquid. The cross‐section of the NCTP on nylon substrate was examined by scanning electron microscope (SEM) (Figure 1f). The overlapped graphene sheets formed the layer‐by‐layer structure of NCTP on the nylon microporous filter membrane. The gap between graphene sheets provided the room for the liquid to be absorbed and slowly released. Therefore, the NCTP‐based chemosensor could reflect the physical state of the measured liquid at various stages.

In many previous studies of graphene‐based chemical‐sensing devices, the chemosensors are usually dipped in a large volume of liquid or exposed to vapor through fumigation. Both approaches ensure that the detector electrode can be completely immersed in the liquid and that the dose or concentration of the sample can be accurately controlled. However, these two approaches may become problematic when they are restricted by the property of the liquid sample, e.g., when the sample volume is very small (≈2 μL) or the sample is nonvolatile. Therefore, when we tested the use of the NCTP as an electronic tongue, we adopted neither of these two methods. Instead, we adopted the dropping method to allow a wider range of application and provide more information of the analytes.

The wetting behavior of water droplet on the NCTP was measured by contact angle titration (Figure S2a, Supporting Information). A certain amount of water was dropped on the surface of NCTP, and heating was constantly applied throughout the entire testing process to ensure uniform temperature. The water droplet diffused on the surface, infiltrated into the graphene film of the NCTP, and then slowly evaporated. The CMC‐modified graphene film was hydrophilic and allowed the water droplet to penetrate slowly. Figure S2b (Supporting Information) shows that the relative resistance of the NCTP escalated rapidly when the added water droplet infiltrated the graphene film, and decreased rapidly as the droplet evaporated. First‐order difference was calculated to determine the start of waveform, and an exponential smoothing was performed to offset the error in the resistance measurement. The start of the waveform was determined as the point where the first‐order difference rose significantly. A group of descriptive parameters (Table S1, Supporting Information) was extracted from the measured curve to describe the waveform (including Maximum, Front‐half‐peak‐width (FHPW), Slope, Re‐slope, and Difference. See the Supporting Information for details).

Liquid sensing by graphene is based on several underlying processes, such as field effect,^21^, ^37^ supporting‐material swelling,^27^, ^39^ hydrogen bonding,^41^, ^42^ etc. The NCTP‐based liquid sensing mainly involves supporting‐material swelling and hydrogen bonding (Figure 1g). Because the capillary force accelerates the flow of liquid into the microchannel of NCTP, the graphene film and filter membrane will swell and generate a relatively small resistance change. In addition, since hydrogen bonds are formed between the polar solvent and the NCTP, the localized inhomogeneous dipole fields near graphene can affect the carrier density. It is worth noting that the cleaned graphene sheets show little or no response to most solvents,^35^ and their large response to polar molecules results from the covalent functionalized oxygen‐containing groups in NCTP.

The swelling mechanism of our prepared NCTP was studied by immersing it in water to fully wet and characterizing it with a step profiler (Figure S2c, Supporting Information). In the presence of water, the in situ film thickness of the NCTP depended on its substrate. Three kinds of microporous filter membranes, i.e., nylon, polytetrafluoroethylene (PTFE), and glass fiber (GF), were measured, and they deformed to varying extent. Applying stress to the NCTP surface could change its electrical resistance (Figure S3a, Supporting Information). The relative resistance change of the NCTP with different substrates was quite different in the presence of water (Figure S2d, Supporting Information). Positive correlation was found between the strength of the resistance change and the degree of deformation, indicating that deformation was one of the main factors affecting the resistance change. In particular, swelling was the main reason for the small relative resistance change of NCTP in the presence of nonpolar organic solvents. A positive correlation also existed between the initial resistance and the Maximum value of the NCTP with different substrates (Figure S3b, Supporting Information). Therefore, the swelling of substrate was one of the main causes of resistance change.

The hydrogen bonding mechanism was examined by calculating waveform parameters in sensing different organic solvents. The physical and chemical parameters of the tested organic solvents are summarized in Table S2 (Supporting Information). A positive correlation was found between the Maximum value and the polarity of the organic solvent, as shown in Figure S2e (Supporting Information). The more polar organic solvent, which was more capable of forming hydrogen bonds, gave stronger resistance response. The sensitive response of our NCTP to polar solvents was due to the formation of hydrogen bonding between the analyte and the functional groups of the NCTP. The hydrogen‐bonded polar molecules could then form a random array of localized inhomogeneous electrostatic fields near graphene, and these random dipole potentials could form random trapping of dipoles near the surface of graphene, thus leading to a significant change in resistance.^43^, ^44^

The response waveform was also affected by many other factors, such as the liquid evaporation rate. The faster the liquid evaporated, the sooner the response waveform peaked. Many factors affected the evaporation rate of liquid, among which the heat of vaporization (kJ mol^−1^) was the most important. Figure S2f (Supporting Information) shows that the heat of vaporization was positively correlated with the FHPW. In addition, the viscosity of the analytes was also positively correlated with FHPW (Figure S2g, Supporting Information), since highly viscous liquid penetrated more slowly into the NCTP and gave larger FHPW.

### 2.2 Identifying Different Liquids by Pattern Recognition

**Figure 2:**
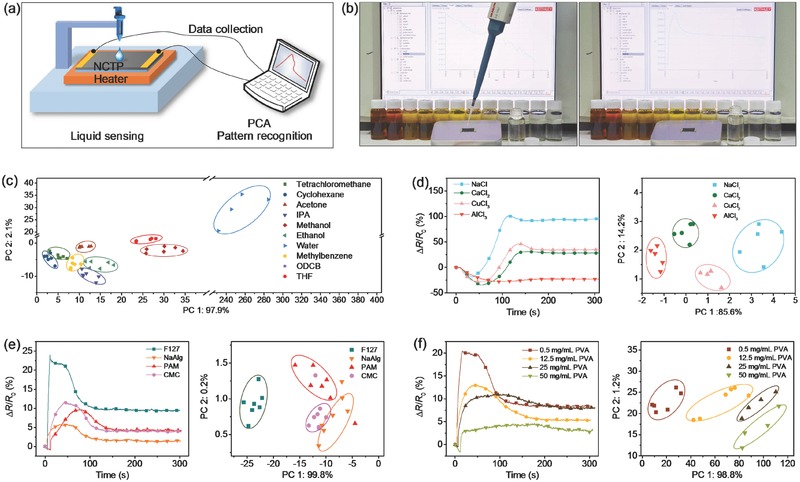
a) Schematic and b) photograph of the dripping test for liquids identification. c) Organic solvents, d) saline solutions, e) polymer solutions, and f) PVA solutions of different concentrations. 2D representations of PCA plots show clear clustering of different analytes.

**Figure** **2**a,b shows the schematic and photograph of the dripping test. As mentioned above, descriptive parameters were first extracted from the resistance change curve of the NCTP through pattern recognition, and the dimension of the data structure was reduced by PCA algorithm.^45^ To reduce the effect of intragroup variances, each data point was first discounted by the distance between the data point and the center point of the corresponding data group. Eleven key descriptive parameters were applied in the pattern recognition to identify the statistical separation among the mean values. Table S1 (Supporting Information) lists the details on extracting the descriptive parameters and the data set. In the mathematical model, multidimensional data were reduced to a single data point on a new principal component (PC) axis. Since the characteristic peak of the NCTP waveform was quite different for dissimilar liquid, different descriptive parameters were selected as the data set of PCA to better distinguish similar liquids.

#### 2.2.1 Analysis of Organic Solvents and Electrolyte Solutions

Because of its detection mechanism, the NCTP is particularly suitable for sensing liquids that differ in polarity, viscosity, heat of vaporization, or conducting property. The PCA diplot in Figure 2c shows that the NCTP could differentiate various organic solvents with high selectivity. The detection of these organic solvents made use of the PTFE‐based NCTP, because PTFE was more chemically stable than nylon and could withstand organic solvents very well (Figure S4, Supporting Information).

The aqueous solutions of metal salts were also tested. The solutions of four metal chlorides of the same concentration (1 mol L^−1^) gave different responses on the NCTP (Figure 2d, left panel). Compared with the detection of organic solvents, the most notable difference was that the relative resistance slumped when the salt solution was added to the NCTP. This initial depression in resistance was because the ions in the solution enhanced the electron transfer. When the water eventually evaporated, these free ions lost their ability to assist in conducting electricity. Among the tested salt solutions, the solution of sodium chloride had the fewest number of ions, and thus its response waveform was the most similar to that of water and had the smallest initial resistance decrease. Figure 2d (right panel) shows that all samples of the metal salt solutions were correctly classified. The calculated principal components were linear combinations of the original parameter set with 11 descriptive parameters.

In addition, the aqueous solution of different kinds of polymer (sodium alginate (NaAlg), polyacrylamide (PAM), carboxymethylcellulose sodium (CMC) and F‐127) (2.5 mg mL^−1^) was also tested (Figure 2e). The PCA biplot shows a clear clustering of different polymer solutions. The first two principal components (PC1 and PC2) explained almost 99% of the parameter variance. Furthermore, the response waveforms of the aqueous solutions of polyvinyl alcohol (PVA) are shown in Figure 2f (left panel). The viscosity of PVA solution increased with its concentration. Raising the concentration of PVA decreased the polarity of the solution and thus resulted in lower maximum relative resistance in the NCTP sensing waveform. At the same time, as the viscosity of polymer solutions increased, the infiltration rate of the solution into NCTP decreased, which led to the increase of FHPW and Difference (Figure S5, Supporting Information). Data processing by PCA for the NCTP sensing results of the PVA solutions gave two principal components, whose scores are plotted in Figure 2f (right panel). The first and the second PC explained 98.8% and 1.2% of the parameter variance, respectively. The PVA solutions of different concentrations were identified and distinguished unambiguously.

#### 2.2.2 Analysis of Five Tastes

**Figure 3:**
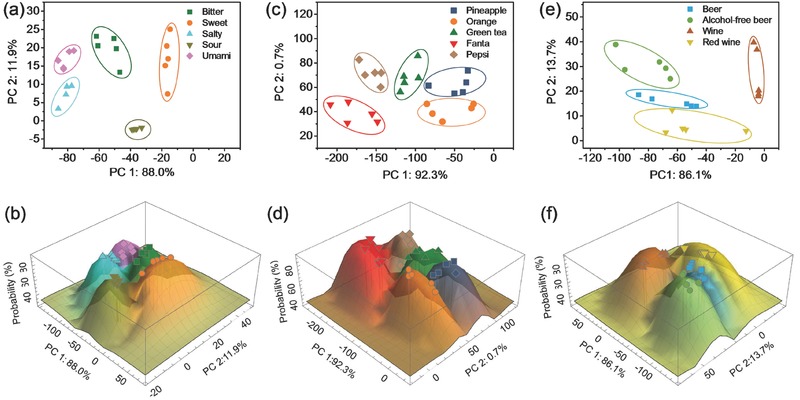
Liquid recognition with NCTPs. a,b) Five tastes. c,d) Soft drinks. e,f) Beers and wines.

The NCTP was tested as a flexible electrical tongue to distinguish the five tastes, namely bitter, sweet, salty, sour, and umami. The corresponding sample liquids were balsam pear lixivium, aqueous solution of sugar, aqueous solution of table salt, rice vinegar, and aqueous solution of monosodium glutamate, respectively. The five tastes were well distinguished from each other (**Figure** **3**a). As expected, the waveform of the salty taste showed the lowest maximum relative resistance because the amount of electrolyte was the most abundant in its solution (Figure S6a, Supporting Information). The difference between the relative resistance at the start and at the end of the waveform was the smallest for the sour taste, possibly because compared with the solutions of other tastes, rice vinegar contained the most volatile solute and left the least amount of solute on the graphene film after evaporation. Here, we introduced the Probability (Figure 3b), and the probability value stands for the probability that the classification is correct. For example, assume the electrical response of liquid X could be translated to a data point *x* through PCA method and parameters description. The Probability value is higher (i.e., the liquid has a higher probability of being bitter) when the point *x* is closer to the center of the “Bitter” region. The test results proved that the NCTP attained high recognition accuracy for the five tastes.

#### 2.2.3 Analysis of Soft Drinks and Liquors

Figure 3c shows the test results of several soft drinks, which were easily discriminated from each other by the NCTP sensing system. The soft drink Fanta separated the most from the other four soft drinks (pineapple juice, orange juice, green tea, and Pepsi coke), most likely because Fanta contained a significantly higher amount of sugar and gum (Figure S6b, Supporting Information). The NCTP system made highly accurate recognition even for similar soft drinks (Figure 3d). All tested beverages could be discriminated with single substrate NCTP system, except Sprite (Figure S7, Supporting Information). We then applied a combined NCTP system consisted of NCTPs with Nylon, PTFE, and GF substrates to detect liquids with similar properties (e.g., green tea, Pepsi, and Sprite). The NCTP system with different substrates could provide data set of higher‐dimensional parameters. Hence, the dimensionality reduction was optimized, which could improve the pattern recognition performance.

Figure 3e and Figure S6c (Supporting Information) show the test results of four different liquors, i.e., beer, nonalcoholic beer, Chinese white wine, and red wine. The PCA results show that they were very well distinguished from each other. In detecting these different liquors, NCTP also attained high recognition accuracy (Figure 3f). However, beers of different brands could not be discriminated by NCTP (Figure S8, Supporting Information), possibly because all beers had similar polarity and viscosity. As a nonspecific liquid sensor, NCTP cannot readily identify particular components in the analytes.

### 2.3 Test of Food Quality

#### 2.3.1 Quality Inspection of Alcohol

**Figure 4:**
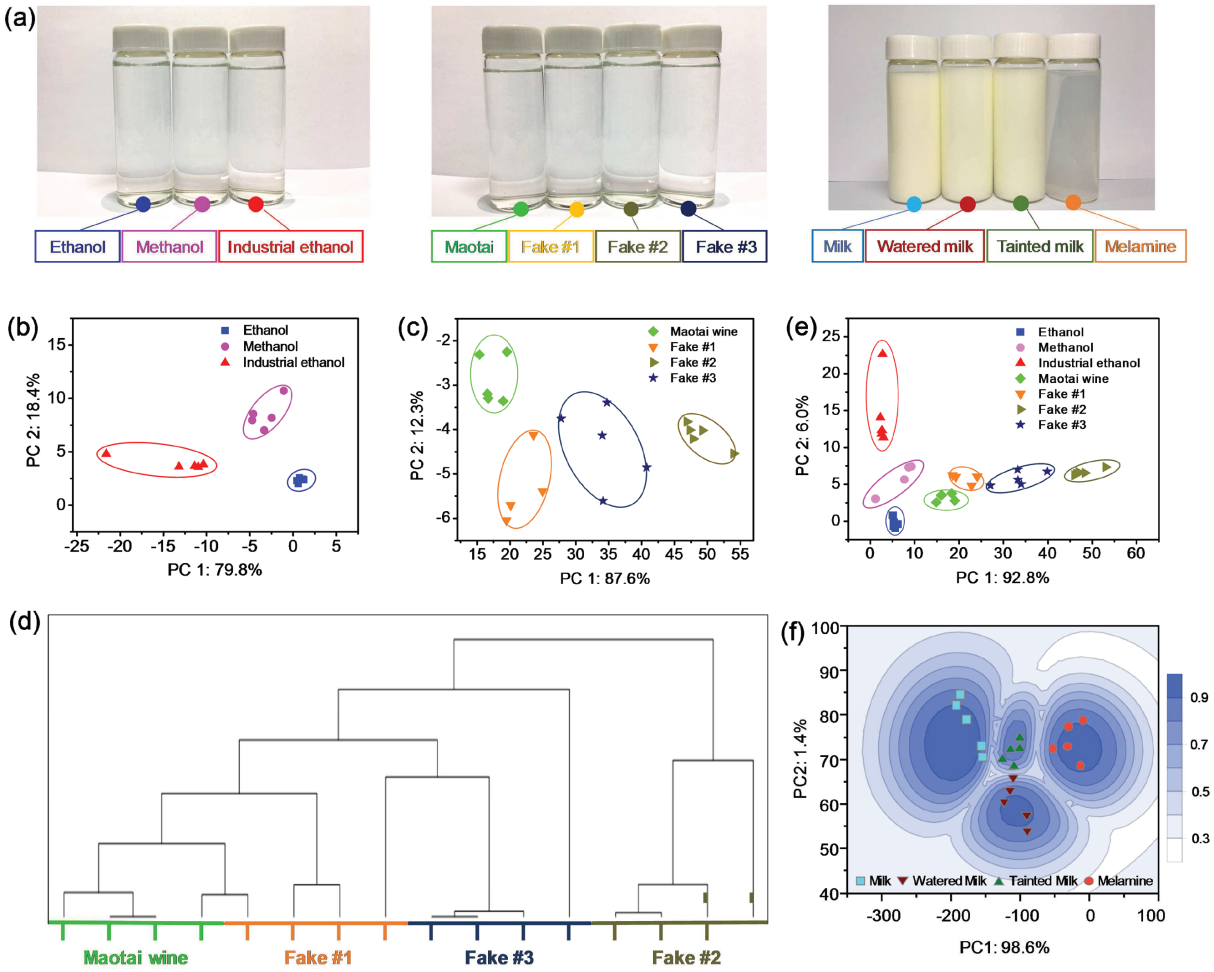
Quality inspection with NCTPs. a) Photographs of tested liquids. PCA plots (calculated based on DataSet 1 in Table S1 in the Supporting Information) for b) ethanol, methanol, and industrial ethanol, c) Maotai wine, watered Maotai (Fake #1), and blended wine made from ethanol (Fake #2), and industrial ethanol (Fake #3). d) HCA plot. e) Genuine liquors identified based on the PCA results. f) Contour map of milks detection.

Taste is the primary indicator of the quality of wine and also one of the most important considerations when the consumer decides to purchase. Various counterfeits of luxury wine circulate in the market because their producers are enticed by the huge profits that can be reaped through such fraud. Some counterfeits produced by cheap industrial alcohol may even contain methanol, which is toxic and, if ingested, can cause blindness or even death. Methanol, ethanol, and industrial ethanol are all colorless liquid and cannot be distinguished from appearance (**Figure** **4**a, left panel). However, they can be easily identified by the NCTP, because methanol is more polar and less viscous than ethanol. The three alcohols are very well separated on PCA score plot (Figure 4b) by adopting appropriate DataSets in Table S1 and Figure S9 (Supporting Information), and we used this as the basis for detecting fake wine. We tested four kinds of wine, i.e., genuine liquor Maotai, watered Maotai, blended wine made from ethanol, and blended wine made from industrial alcohol (alcohol concentration = 53 vol%) (Figure S10a,b, Supporting Information). They all have identical appearance (Figure 4a, middle panel), but the fake wine samples could not escape detection by the NCTP (Figure 4c,d). The genuine liquor and the fake wine appeared in distinct clusters on the hierarchical cluster analysis (HCA) and PCA plots (Figure 4d,e).

#### 2.3.2 Quality Inspection of Milk

Milk is one of the best sources of high‐quality protein and is thus an important food for human health. The content of protein is a key indicator of the quality of milk. The Kjeldahl method can quantitatively determine the amount of organic nitrogen in substances,^46^ and it is applied universally to assess the quality of milk because in milk, only protein contains nitrogen. However, this method failed when unethical merchants doped melamine into milk. Melamine is a chemical that has a high nitrogen content, but it is harmful to human health and can significantly increase the risk of causing kidney stones. Using the NCTP sensing system, we analyzed genuine milk, watered milk, melamine aqueous dispersion, and milk tainted with melamine. The photograph in Figure 4a (right panel) shows that the poisonous milk could not be distinguished by appearance. However, the strongly polar melamine gave a unique response peak in its relative resistance waveform (Figure S10c, Supporting Information), making it possible to detect the tainted milk by NCTP. In the contour map of PCA plots of the milk samples (Figure 4f), the first two principal components (PC1 and PC2) explained almost 100% of the descriptive parameters. Accordingly, a contour map of pattern recognition results was given, in which the depth of color represents the Probability value. The tainted milk and the watered milk were both very clearly separated from the genuine milk, which proves the utility of the NCTP liquid‐sensing system. Each kind of liquid was tested five times, and the dispersion of data points in PCA plots reflected errors and uncertainty correlated with the original resistance of NCTP (Figure S12, Supporting Information). The high sensitivity of NCTPs and the pattern recognition algorithm helped to minimize the errors. Overall, the NCTP‐based liquid‐sensing system could distinguish a variety of liquids repeatedly, which proved the good reproducibility of the system.

In summary, we have fabricated a flexible multifunctional NCTP through ion‐assisted vacuum filtration. The NCTP could work as a flexible electrical tongue for the qualitative analysis of multicomponent solutions. This NCTP‐based liquid‐sensing system was particularly suitable for detecting liquids of different polarity, viscosity, vaporization heat, or conductive property, but was not suitable for analyzing volatile organic solvents that have low vaporization heat. The NCTP successfully discriminated different metal salt solutions and polymer solutions, and it also identified five different tastes, soft drinks, and alcoholic liquors. The NCTP could also identify fake wine and tainted milk and thus showed great potential in the quality assurance of foodstuff. The NCTP‐based liquid‐sensing system provides reliable and rapid response and is suitable for a wide range of applications. In the future, it may be possible to implement wearable, flexible, intelligent liquid analysis systems with disposable NCTPs in smart bracelet, industrial quality control, and electrical tongues in robots. It remains an open question for future research to see if loading functional materials, such as small molecule selectors,^29^ metal,^47^, ^48^ or metal oxide,^49^, ^50^ to the NCTP may enable more specific recognition.

## 3 Experimental Section

*Preparation of Graphene Conductive Ink*: Multilayer graphene (XF001W, XF Nano) was dispersed in DI water at the concentration of 0.1 mg mL^−1^, and 0.25 mg mL^−1^ sodium carboxymethylcellulose (Shanghai Reagent, 200 cP at 20 g L^−1^) was added to improve the dispersion of graphene in water. The mixture was sonicated with a contact‐free sonication system (50 W) for 3 h. The graphene conductive ink was then filtered (1000 mesh) to remove the undispersed graphene.

*Preparation of NCTPs*: The CMC‐modified graphene conductive ink was vacuum filtered under 1 MPa for 2 min with a membrane filter. The ions in CMC allowed NCTP to form a relatively smooth and dense film.^51^ Membrane filters (0.22 μm in pore size, 50 mm in diameter) were purchased from the Taoyuan company. Several materials were selected as the filter membrane and thus the substrate for the NCTP. The nylon membrane filter was suitable for making NCTP that detected aqueous solution, as it is hydrophilic and can tolerate some organic solvents. The PTFE membrane filter was more suitable for making NCTP that detected organic solvents because it is chemically inert. The polymer membranes could be cut easily by knife and scissors. The electrode of the NCTP was prepared by template‐assisted thermal evaporation deposition of gold.

*Liquid Test Procedure*: Liquid identification tests were conducted at a constant temperature of 50 °C, to enhance liquid evaporation and improve the detection efficiency. The volume of the test liquid was measured by micropipette tips. The electrode of NCTP was connected by test fixture or silver solution. After a liquid droplet of the sample was applied, nonselective analysis was performed by measuring the resistance change curve of the NCTP and extracting appropriate parameters from the curve through pattern recognition.

*Liquid Sample Preparation*: Electrolyte (NaCl, CaCl_2_, CuCl_2_, and AlCl_3_) solutions had the same concentration of 1 mol L^−1^. The tested polymers included F‐127 (Sigma), NaAlg (Sinopharm chemical reagent), PAM (Sinopharm chemical reagent, CAS: 9003‐05‐8), and CMC (Shanghai Reagent, 200 cP at 20 g L^−1^). The aqueous solutions of the polymers had the same concentration of 2.5 mg mL^−1^. The tested liquids of different tastes were balsam pear lixivium (balsam pear juice), sugar solution (10 g L^−1^), edible salt solution (10 g L^−1^), rice vinegar (9% white rice vinegar from Hengshun Co., Ltd.), and monosodium glutamate aqueous solution (0.5 mol mL^−1^). The industrial alcohol used in the quality inspection was a mixture of ethanol and methanol with a volume ratio of 96:4. The wine detection was based on the Maotai liquor (Guizhou Maotai Distillery, Hongxijiang, alcohol concentration: 53 vol%). The volume ratio of wine and DI water in watered Maotai was 2:1. Fake wines were prepared by mixing water with ethanol or industrial ethanol (alcohol content: 53 vol%). The tested milk samples included liquid milk (Sanyuan Foods Co., Ltd., pure milk) and watered milk (the volume ratio of milk and water was 2:1). The concentration of melamine (Innochem, 99%) in aqueous suspension was 6 g L^−1^. The melamine powder was mixed with liquid milk to prepare tainted milk (3.08 g L^−1^ melamine).

*Pattern Recognition*: PCA converted a data set with possible correlated variables into a set of values with linearly uncorrelated variables (called principal components), and examined the principal components' contributions to the total variance of the data set. By eliminating the principal components with little contribution to variance, the dimension of the data set could be significantly reduced. HCA is a method for classifying data and building a hierarchy of clusters.^45^ In this work, the strategy for HCA was to calculate the distances between the data points of PC 1 and then cluster the near distance points to the same layer.

*Characterizations*: The microtopography of NCTP was characterized by scanning electron microscope (SEM, LEO 1530, 5 kV). The resistance response was measured in real time with a digital universal meter (Keithley 2401) at a test step of 1 ms. The applied voltage was 10 V, and the testing current was around a few milliampere. Raman spectroscopy of NCTP was measured on a LabRAM HR Evolution instrument (He‐Ne laser excitation at 514 nm, 5 mW). The thickness of the NCTP was tested on a step profiler (Bruker corporation, DektakXT).

## Conflict of Interest

The authors declare no conflict of interest.

## Supporting information

Supplementary
