# Supplementary material for: Rapid Liquid Recognition and Quality Inspection with Graphene Test Papers
Source: Glob Chall. 2017 Jul 10;1(6):1700037. doi: 10.1002/gch2.201700037 (PMC6607296; doi:10.1002/gch2.201700037)
Supplement: Supplementary file 1 — Supplementary [file GCH2-1-1700037-s001.pdf]

Copyright WILEY-VCH Verlag GmbH & Co. KGaA, 69469 Weinheim, Germany, 2017.

Supporting Information

for *Global Challenges,* DOI: 10.1002/gch2.201700037

Rapid Liquid Recognition and Quality Inspection with Graphene Test Papers

*Xin Jiang, Tingting Yang, Changli Li, Rujing Zhang, Li Zhang, Xuanliang Zhao, and Hongwei Zhu**

Copyright WILEY-VCH Verlag GmbH & Co. KGaA, 69469 Weinheim, Germany, 2017.

Supporting Information

Rapid Liquid Recognition and Quality Inspection with Graphene Test Papers

*Xin Jiang, Tingting Yang, Changli Li, Rujing Zhang, Li Zhang, Xuanliang Zhao, and Hongwei Zhu**

This file includes:

S1. PCA method and Parameters Description
S2. Supplementary Tables and Figures

S1. PCA method and Parameters Description

The principal component analysis (PCA) converted a data set with possible correlated variables into a set of values with linearly uncorrelated variables (called principal components), and examined the principal components' contributions to the total variance of the data set [S1]. By eliminating the principal components (PCs) with little variance contributions, the dimension of the data set could be significantly reduced. The PCA algorithm was performed by the following steps:

1) Given a data matrix **X**, computed its covariance matrix $\mathbf{S} = \mathrm{Cov}(\mathbf{X})$.

2) Calculated the eigenvalue-eigenvector pairs $(\lambda_1, \boldsymbol{e}_1), (\lambda_2, \boldsymbol{e}_2), \cdots, (\lambda_n, \boldsymbol{e}_n)$ of the covariance matrix **S**, where $\lambda_1 \geq \lambda_2 \geq \lambda_3 \geq \cdots \geq \lambda_n$.

3) Calculated the values of principal components. The ith principal component was $\mathrm{PC}_i = \mathbf{X} \cdot \boldsymbol{e}_i$, and its contribution to the total variance was $\lambda_i / \sum_{k=1}^{n} \lambda_k$.

The PCA algorithm was empolyed to find out the linear combination of parameters that are most effective in distinguishing liquids, and the calculated PCs represent the linear combinations of the orginal parameter set with 11 descriptive parameters.

Before performing the PCA algorithm, each data point was discounted by a distance-related weight, in order to reduce the effect of intra-group variances. The distance between each data point and the center point of the corresponding data group was measured, and a portion of data in each group was chosen according to the descending order of all the

distances. The weight *w* defined the proportion of the data set that was chosen. The procedure was called weighted principal component analysis (WPCA).

The classifier applied in pattern recognition was support vector machine (SVM) classification [S2,S3]. The probability is caculated from PC1 and PC2 through SVM. All of the above calculations were performed with Wolfram Mathematica.

Maximum is defined as the maximum value in the waveform of the relative resistance.

Front-half-peak-width (FHPW) is the time difference between the waveform starting time and its peak time.

Slope represents the ratio of *Maximum* to *FHPW*, and *Re-slope* is the inverse of *Slope*.

Effect-ratio is denoted as the ratio of the relative resistance at time $p+\alpha\times FHPW$ to that at time $p-\alpha\times FHPW$, where p is the peak time and α is the ratio parameter. The value of α in *Effect-ratio1*, *Effect-ratio2*, *Effect-ratio3*, *Effect-ratio4*, *Effect-ratio5* is 0.1, 0.2, 0.4, 0.8 and 1, respectively.

Difference is denoted as the difference between the relative resistances at the waveform start time and the end time.

Start-difference is denoted as the first order difference of the relative resistance at the waveform start time.

Peak-value is the difference between *Maximum* and the relative resistance at the start time of the waveform.

S2. Supplementary Tables and Figures

Table S1. Parameters for PCA calculations.

	FHPW	Front-slope	Re-slope	Maximum	Peak-value	Difference	Effect-ratio1	Effect-ratio2	Effect-ratio3	Effect-ratio4	Start-difference
Organic solvents	√		√	√	√			√	√		
Saline solutions						√	√	√	√	√	
Polymer solutions (content)				√	√		√	√	√	√	
Polymer solutions (concentration)	√	√		√		√		√		√	
Five flavor	√	√		√	√	√	√	√	√		
Soft drinks	√		√	√		√					√
Liquors	√		√	√		√	√	√			
Beers	√		√					√	√	√	
Fake ethanol			√	√	√		√	√	√	√	
Fake wine (DataSet 1)			√	√	√		√	√	√	√	
Fake wine (DataSet 2)					√		√	√	√	√	
Fake wine (DataSet 3)	√	√			√		√	√			
Fake milk	√	√		√	√	√	√	√	√		
Urine	√	√		√	√		√	√	√		

* "✓" represents the inclusion of the data set of WPCA. As the response waveform of resistivity was affected by liquid parameters, the waveform reflected the liquid characteristics. For example, the viscosity could affect the *front-half-peak-width* of the response waveform. In distinguishing a group of similar liquids, the most accurate results could be obtained with a particular selection of specific parameters.

Table S2. Organic analytes parameters tested with NCTP sensing system*.

	Molecular weight (g/mol)	XLogP3 (a.u.)	Boiling point (°C)	Density (g/cm^3)	Viscosity (cP)	Heat of vaporization (kJ/mol)	Polarity index (P')	Hydrogen bond count (a.u.)
Acetone	58.08	-0.1	56.1	0.78	0.32	30.99	5.1	1.34
Acetonitrile	41.05	0	81.6	0.8	0.35	29.85	5.8	1.95
Chloroform	119.37	2.3	61.2	1.49	5.63	29.64	4.1	0
Cyclohexane	84.16	3.4	80.7	0.8	0.98	33.06	0.2	0
Ethanol	46.07	-0.1	78.3	0.79	1.07	42.32	4.3	1.71
Ethyl acetate	88.11	0.7	77.1	0.9	0.42	35.60	4.4	2.04
Ethylene glycol	62.07	-1.4	198.0	1.1	-	-	6.9	3.54
IPA	60.10	0.3	82.3	0.79	2.04	45.39	3.9	1.31
Methanol	32.04	-0.5	64.7	0.79	0.54	37.34	5.1	2.47
Methylbenzene	92.14	2.7	110.6	0.87	0.56	38.01	2.4	0
NMP	99.13	-0.5	202.0	1	1.65	52.82	6.7	1.01
ODCB	147.00	3.4	180.1	1.3	1.32	45.72	2.7	0
Tetrachloromethane	153.81	2.8	76.8	1.59	2.03	34.50	1.6	0
THF	72.11	0.5	65.0	0.89	0.53	29.56	4	1.23
Water	18.02	-0.5	100.0	1	0.89	40.67	10.2	5.55

*Data taken from: [i] https://pubchem.ncbi.nlm.nih.gov/; [ii] https://labchemicals-honeywell.com/

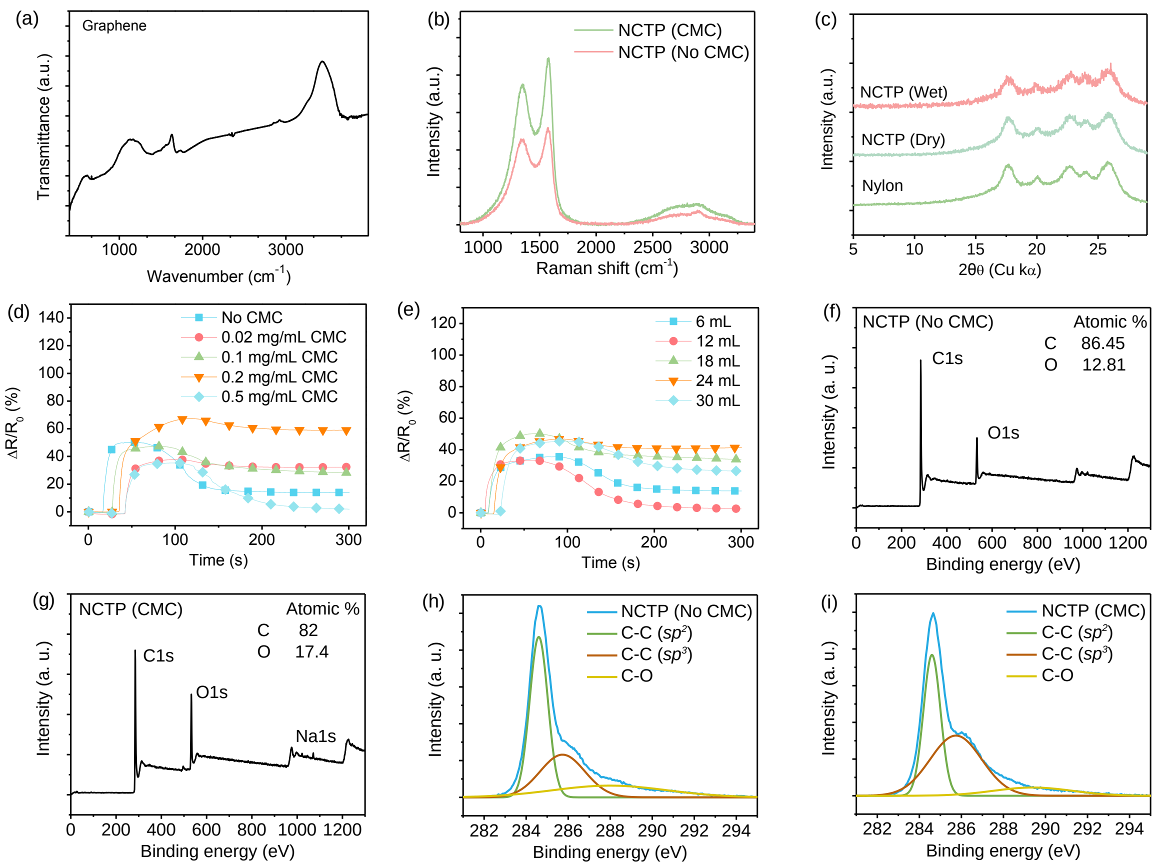

Figure S1. (a) Absorption spectrum of graphene containing functional groups. (b) Raman spectra of pristine and CMC modified NCTPs. (c) XRD patterns of the nylon substrate and NCTPs in dry (humidity: ~40%) and fully wet states, respectively, demonstrating the structural instability of nylon-based-NCTP in water. (d) Response waveforms of NCTPs with different concentration of CMC (in 5 μL ethanol). (e) Response waveforms of NCTPs with different volume of graphene conductive ink (in 5 μL ethanol). Survey XPS spectra of (f) pristine and (g) CMC modified NCTPs. Deconvoluted high resolution C1s spectra of (h) pristine and (i) CMC modified NCTPs. The oxygen content increased apparently for CMC modified NCTP. The C1s XPS spectrum of pristine NCTP reflected the presence of three types of carbon bonds: C-C (sp^2), C-C (sp^3) and C-O at 284.6, 285.73, 287.98 eV. The bands in spectrum of CMC modified NCTP were C-C (sp^2), C-C (sp^3) and C-O at 284.6, 285.73, 289.4 eV.

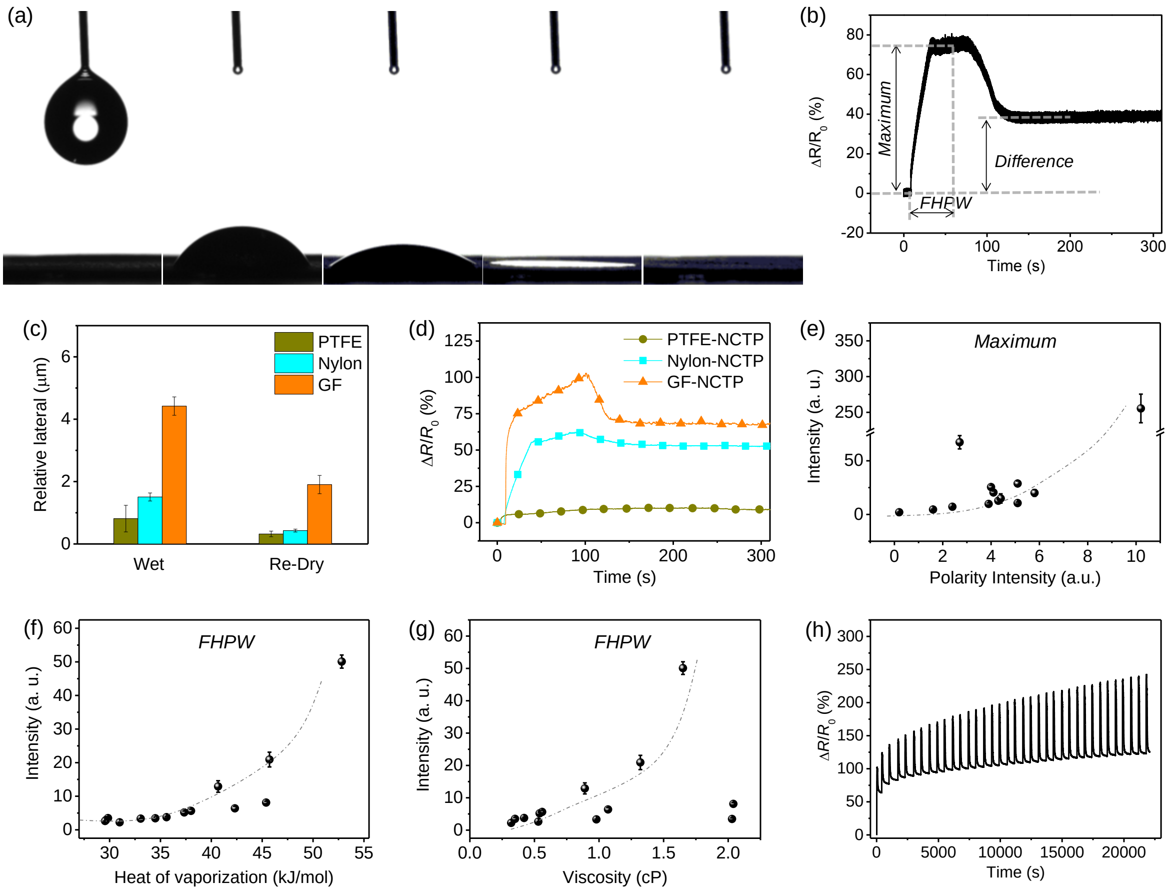

Figure S2. Parameters extraction and pattern recognition by PCA method. (a) Formation, infiltration and evaporation of a water droplet (4.5 μL, 50°C). (b) Response waveform of nylon-based NCTP to the water droplet. (c) Relative thickness changes of NCTPs with different substrates. (d) Response waveforms of NCTPs with different substrates to a 3 μL water droplet. Correlations between (e) *Maximum* and polarity, (f) *FHPW* and the heat of vaporization, (g) *FHPW* and viscosity of the detected liquids. (h) Repeated cyclic measurements of ethanol (~10 μL).

The NCTP was also tested in wetting/drying cycles with ethanol (**Fig. S2h**). The response of electrical resistance stabilized after several cycles. The relative resistance increased slightly after each detecting cycle, which was also observed in previous studies [S4-S6]. It is normal that the resistance of NCTP could not return exactly to the original value, because the incomplete evaporation of the liquid may leave some residual analyte on the NCTP. Note that the resistance recovered almost instantaneously when ethanol was removed. It proves that no chemical bond was formed between the analyte and the graphene sheets [S7].

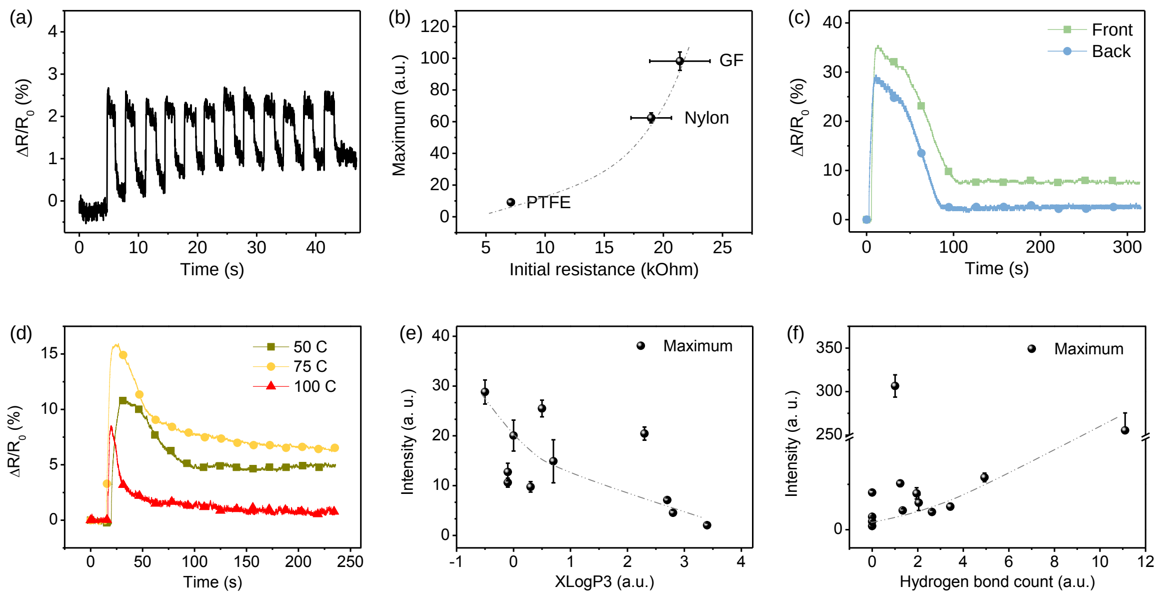

Figure S3. (a) Relative resistance change of NCTP upon finger pressing. (b) Correlation between the initial resistance and *Maximum* for NCTPs on different substrates. (c) Response waveforms of NCTP to water droplets (2 μL) added to the front side and back side, respectively. (d) Response waveforms of NCTP to ethanol droplets (5 μL) at different testing temperatures. (e) Negative correlations between *Maximum* and XLogP3 (calculated hydrophobic parameters). (f) Positive correlation between *Maximum* and calculated hydrogen bond count (in 2 μL solvent).

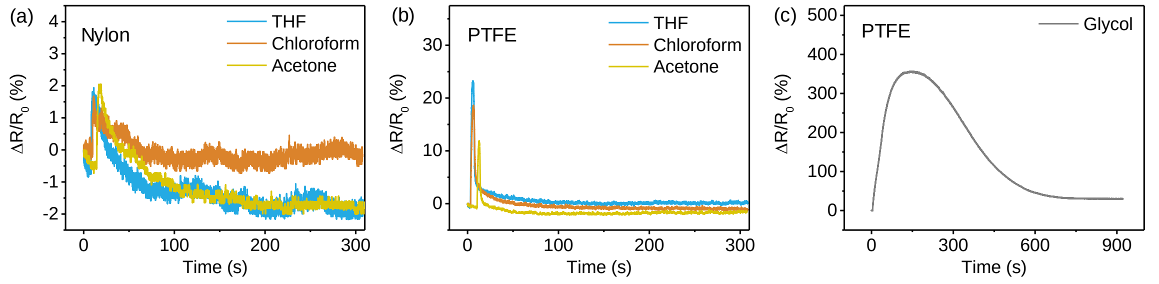

Figure S4. Organic solvents detection with (a) nylon-based and (b) PTFE-based NCTPs. The chemical stability of PTFE suppressed the signal noise during data acquisition. (c) Response waveform for glycol, a high-viscosity and low-polarity component. For liquids that are difficult to volatilize, the time to obtain a complete waveform increased significantly.

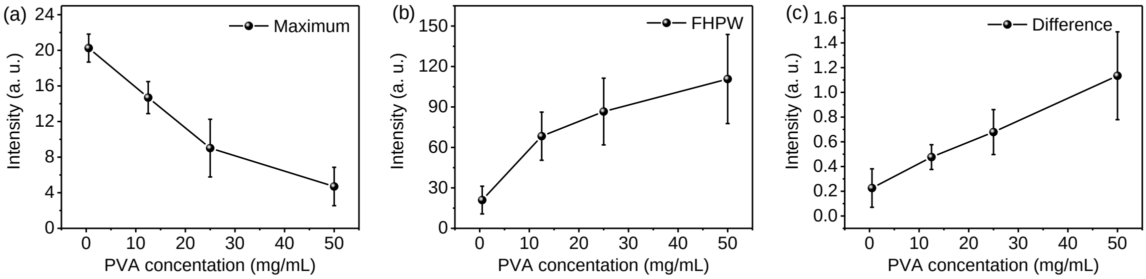

Figure S5. Description parameters effected by PVA concentration. (a) Maximum. (b) FHPW. (c) Difference.

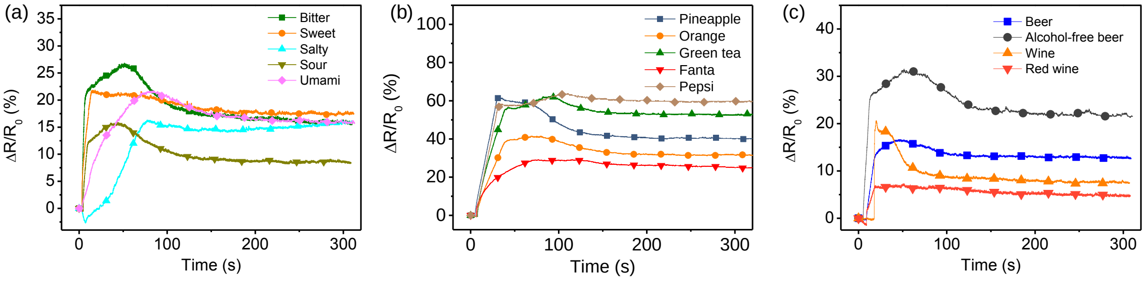

Figure S6. Response waveforms of NCTP for different liquids. (a) Five tastes, including bitter, sweet, salty, sour, umami. (b) Soft drinks, including sport drinks of pineapple flavor and orange flavor, green tea, Fanta and Pepsi. (c) Liquors, including beer, alcohol-free beer, wine and red wine.

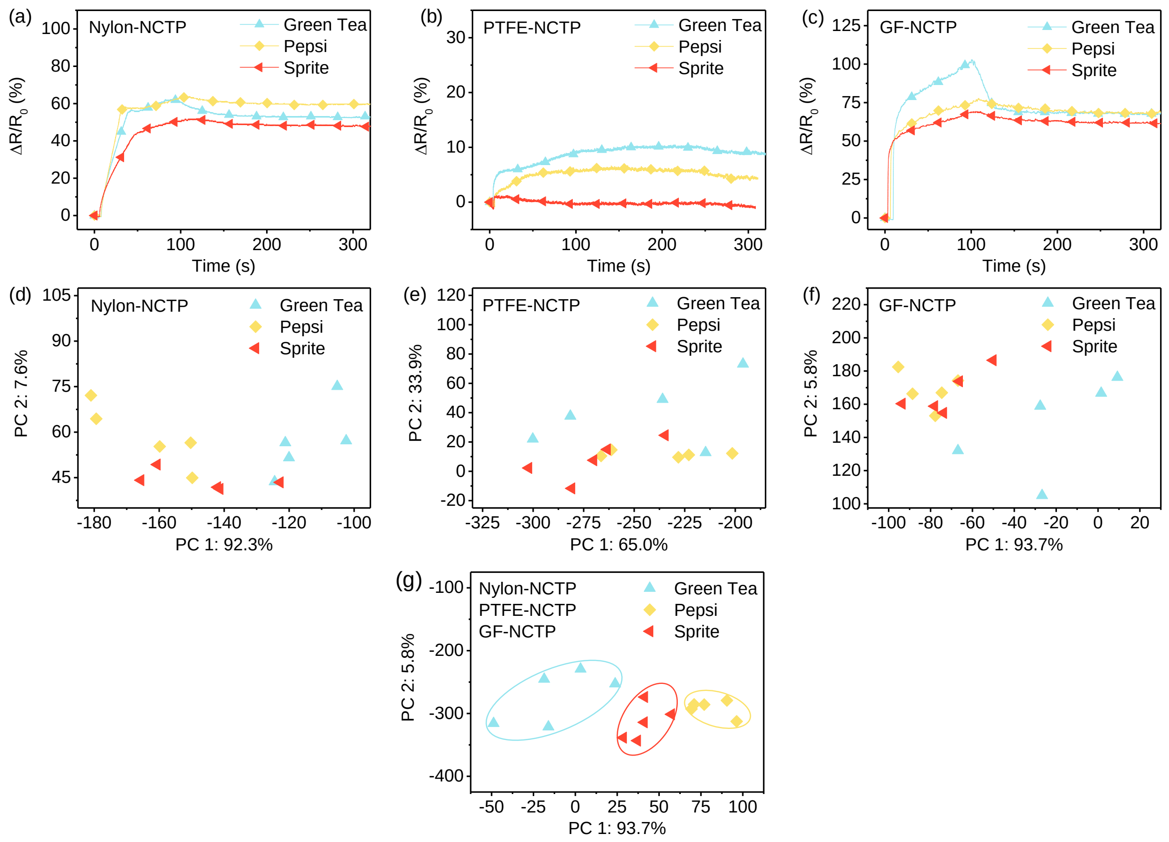

Figure S7. Detection of soft drinks (green tea, Pepsi and Sprite) by combining NCTP systems with different substrates (Nylon, PTFE and GF). (a-c) Resistance responses. (d-g) Pattern recognition results.

The liquid sensing system was applied in detecting different kinds of soft drinks, including sport drinks of pineapple and orange flavors, Fanta, green tea, Pepsi and Sprite. All tested beverages could be discriminated from each other, except sprite. We further raised a combined NCTP system which was consisted of NCTPs with Nylon, PTFE and GF substrates. The combined NCTP system could be applied in detection of liquids with similar properties, for example, green tea, Pepsi and Sprite. The NCTPs collected the resistance responses of the three beverages (**Fig. S7a-c**), and the liquid sensing system with single substrate was unable to recognize the three liquids. Analysis of signals from three different NCTP had shown a clear clustering of different beverages (**Fig. S7d**). The reason was that the NCTP with different substrates could provide dataset of higher-dimensional parameters. The dimensionality reduction direction was optimized, which could improve the pattern recognition results. Therefore, the combined NCTP liquid sensing system offered a unique advantage in distinguishing similar liquids.

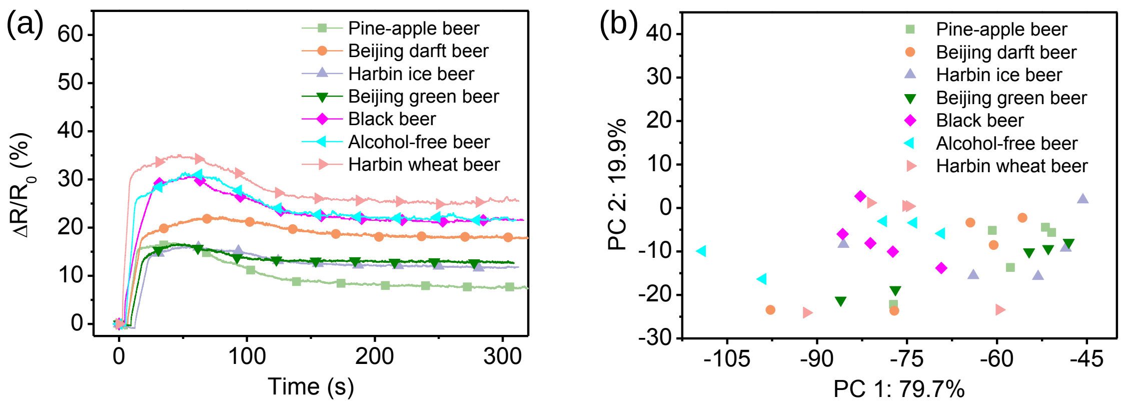

Figure S8. Detection of beers. (a) Response waveforms. (b) PCT plots. The NCTP could not achieve high selectivity for different types of beers. As all tested beers had similar polarity, viscosity and conductivity, the response waveforms were quite similar.

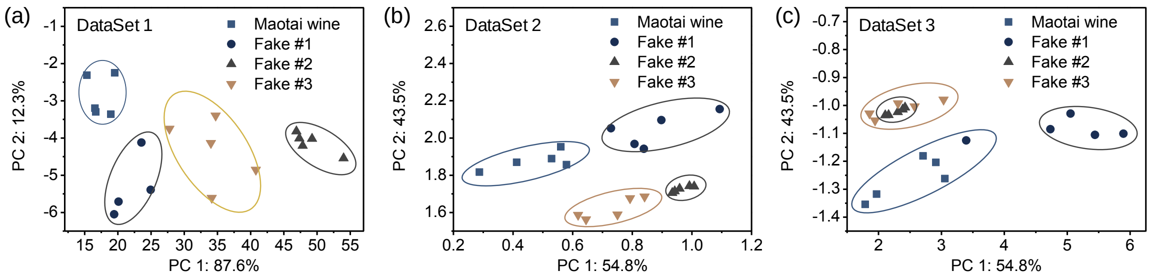

Figure S9. PCA plots for Maotai wine, Fake #1, #2, #3 based on different DataSets in Table S1. (a) DataSet 1. (b) DataSet 2. (c) DataSet 3.

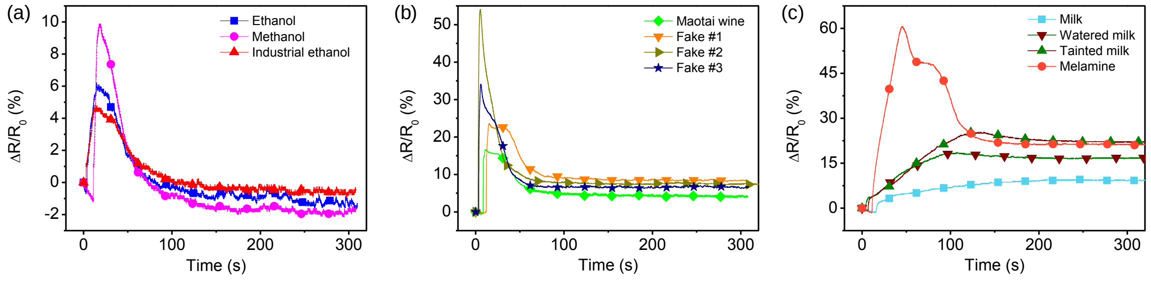

Figure S10. NCTPs for quality testing. (a) Response waveforms of ethanol, methanol and industrial ethanol. (b) Response waveforms of the genuine Maotai wine, watered Maotai (Fake #1), and blended wine made from ethanol (Fake #2), and industrial ethanol (Fake #3). (c) Response waveforms of original milk, watered milk, melamine tainted milk and melamine.

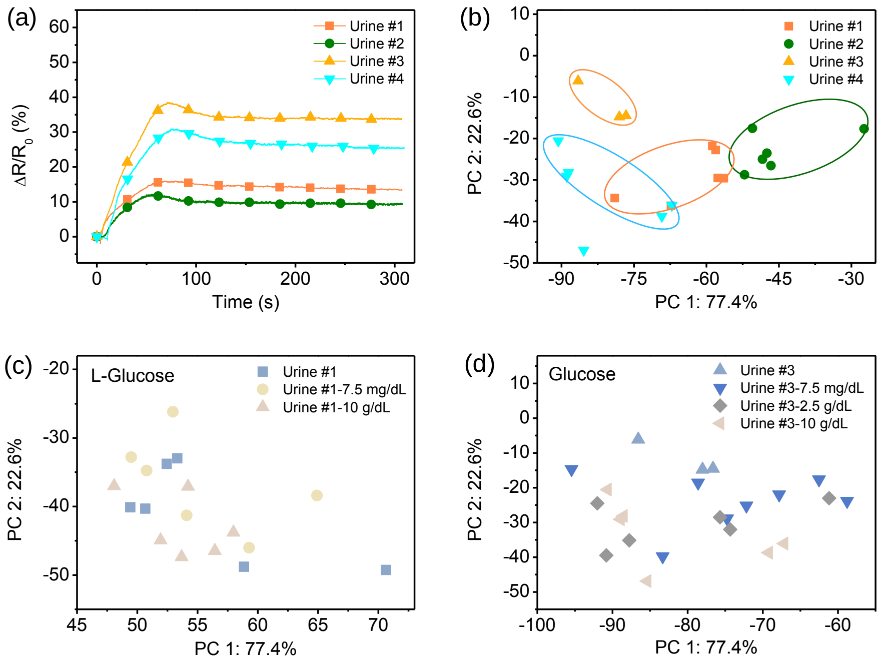

Figure S11. Urine detection for diabetes mellitus. (a) Response waveforms. (b-d) PCT plots for different urine samples.

Urine could reflect the state our health. The everyday diet can affect urine in a number of ways. For example, it has been recommended that one should drink at least 1.5 L water each day to stay hydrated and healthy. Lack of water intake may cause various health problems, including headache, dizziness, fatigue, constipation, *etc.* The state of the urine, most importantly its content of uric acid and urea, is a sensitive indicator of the average daily water intake. In our test, one female volunteer provided three after-meal urine samples (#1, #2, #4), and one male volunteer provided the sample #2. Urine #1 was collected on the period and Urine #3, #4 were collected on the normal time. All samples were collected between 14:00~15:00 after lunch. The body's water content increased after the meal and resulted in the significant difference of the response waveform of the samples. After extraction of parameters and PCA analysis (**Fig. S11a,b**), the sample #2 was well distinguished from other urine samples. We also attempted to analyze the presence of glucose in the urine. However, neither the aldehyde-free L-glucose (**Fig. S11c**) nor the aldehyde-containing glucose (**Fig. S11d**) could be accurately classified, very likely because the response of uric acid sheltered the response of glucose. However, NCTP is an excellent carrier for functional materials, and the specific detection of sugar remains possible if the NCTP could be loaded with certain nanoparticles.

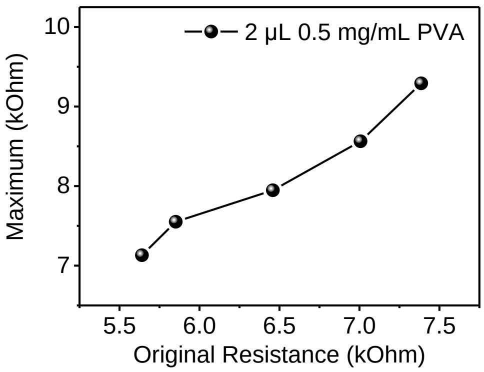

Figure S12. Positive correlations between the original resistance of NCTP and the *Maximum* value of the response waveform.

In the resistivity measurement, systematic error and random uncertainty were unavoidable. In PCA plots of liquid recognition results, the error bars could be considered as the divergence of the data points from same liquid. For the nanocomposite test paper (NCTP) based liquid sensing system, there are two main factors that might cause errors. First, the NCTPs were completely hand-prepared (such as cutting of test paper, electrode connecting). The uncertainty in manual operation led to differences in the original resistance of NCTPs. The original resistance would affect the response waveform of resistivity. For example, for the 0.5 mg/mL PVA aqueous solution, the original resistance value of NCTPs was strongly correlated with the *Maximum* value of the response waveform (**Figure S12**). Second, the liquid dropping process was carried out manually by pipetting, and the random error was inevitably introduced.

However, the NCTP based liquid sensing system still had good reproducibility. In this work, each kind of liquid was tested five times, and it was found that the liquid recognition with NCTPs showed good repeatability and reproducibility. The reason was that the NCTPs possessed high sensitivity and the pattern recognition algorithm helped to reduce the above-mentioned errors. These errors could be further minimized when the preparation of NCTPs and the liquid detection process are fully standardized.

References:

[S1] P. Mukhopadhyay, Multivariate statistical analysis. 2009, World Scientific.
[S2] J. Suykens, et al., Least squares support vector machine classifiers. Neural Process Let. 1999, 9, 293-300.
[S3] D. Lee, et al., A method to predict the impact of regulatory variants from DNA sequence. Nat. Genet. 2015, 47, 955-961.

[S4] T. T. Yang, et al., Interconnected graphene/polymer micro-tube piping composites for liquid sensing. Nano Res. 2014, 7, 869-876.
[S5] S. Z. Guo, et al, 3D printing of a multifunctional nanocomposite helical liquid sensor. Nanoscale 2015, 7, 6451-6456.
[S6] T. T. Tung, et al., Hybrid films of graphene and carbon nanotubes for high performance chemical and temperature sensing applications. Small 2015, 11, 3485-3493.
[S7] X. An, et al., Optical and sensing properties of 1-pyrenecarboxylic acid-functionalized graphene films laminated on polydimethylsiloxane membranes. ACS Nano 2011, 5, 1003-1011.
